# Deep Sequencing Identification of Novel Glucocorticoid-Responsive miRNAs in Apoptotic Primary Lymphocytes

**DOI:** 10.1371/journal.pone.0078316

**Published:** 2013-10-24

**Authors:** Lindsay K. Smith, Arpit Tandon, Ruchir R. Shah, Deepak Mav, Alyson B. Scoltock, John A. Cidlowski

**Affiliations:** 1 Molecular Endocrinology Group, Laboratory of Signal Transduction, NIEHS, NIH, Department of Health and Human Services, Research Triangle Park, North Carolina, United States of America; 2 SRA International, Durham, North Carolina, United States of America; Emory University, United States of America

## Abstract

Apoptosis of lymphocytes governs the response of the immune system to environmental stress and toxic insult. Signaling through the ubiquitously expressed glucocorticoid receptor, stress-induced glucocorticoid hormones induce apoptosis via mechanisms requiring altered gene expression. Several reports have detailed the changes in gene expression mediating glucocorticoid-induced apoptosis of lymphocytes. However, few studies have examined the role of non-coding miRNAs in this essential physiological process. Previously, using hybridization-based gene expression analysis and deep sequencing of small RNAs, we described the prevalent post-transcriptional repression of annotated miRNAs during glucocorticoid-induced apoptosis of lymphocytes. Here, we describe the development of a customized bioinformatics pipeline that facilitates the deep sequencing-mediated discovery of novel glucocorticoid-responsive miRNAs in apoptotic primary lymphocytes. This analysis identifies the potential presence of over 200 novel glucocorticoid-responsive miRNAs. We have validated the expression of two novel glucocorticoid-responsive miRNAs using small RNA-specific qPCR. Furthermore, through the use of Ingenuity Pathways Analysis (IPA) we determined that the putative targets of these novel validated miRNAs are predicted to regulate cell death processes. These findings identify two and predict the presence of additional novel glucocorticoid-responsive miRNAs in the rat transcriptome, suggesting a potential role for both annotated and novel miRNAs in glucocorticoid-induced apoptosis of lymphocytes.

## Introduction

Apoptosis of lymphocytes is critical for the homeostatic balance of the immune system. The escape of lymphocytes from apoptotic constraint results in dire consequences including the development of hematomalignancy and autoimmune disorders. Glucocorticoid hormones are potent inducers of lymphocyte apoptosis [1]. Endogenous glucocorticoids regulate immune development through the elimination of unwanted immature thymocytes during the T-cell selection process [2]. Furthermore, given their aggressive pro-apoptotic properties, synthetic glucocorticoids are a mainstay of hematomalignant chemotherapeutic regimens. 

Glucocorticoids are a class of essential stress-induced steroid hormones regulating cardiovascular, metabolic, homeostatic and immunologic functions. Endogenous glucocorticoids are synthesized and secreted under the control of the hypothalamic-pituitary-adrenal axis in response to stressors, including environmental stress, nociception, and emotion [3]. The pleiotropic effects of glucocorticoids are mediated by the ubiquitously expressed glucocorticoid receptor (GR), which serves as a sensor of environmental stress, mediating the response of the immune system to environmental stress and toxic insult. Glucocorticoid-induced apoptosis of lymphocytes is a multifaceted process, requiring signaling through the GR and the altered expression of apoptotic effector genes [4-6]. Several laboratories have performed genome-wide microarray analysis to delineate the changes in gene expression that modulate glucocorticoid-induced apoptosis. Most notably, the expression of the pro-apoptotic BH3-only Bcl-2 family member Bim is induced by glucocorticoid-treatment in murine lymphoma cell lines, human leukemic cell lines, mouse primary thymocytes, as well as human primary chronic lymphoblastic leukemia and acute lymphoblastic leukemia samples [7-9]. While not the only mechanism involved in this complex process, the upregulation of Bim is likely an important mediator of glucocorticoid-induced apoptosis, as both in-vivo and in-vitro depletion of Bim expression in lymphocytes decreases sensitivity to glucocorticoid-induced apoptosis [10-12]. However, until recently, gene expression analysis of lymphocytes undergoing glucocorticoid-induced apoptosis has largely ignored the examination of non-coding RNAs, or miRNAs. 

MiRNAs are non-coding, ~21mer, single-stranded post-transcriptional regulators of gene expression [13,14]. First discovered in *C. elegans* fifteen years ago, highly conserved miRNAs have now been identified and cloned in plants, *D. melanogaster*, rodents, humans and numerous other species [15-18]. The interaction of a miRNA with mRNA (via imperfect “seed sequence” binding) hinders target mRNA translation while increasing evidence demonstrates that miRNAs can also promote the deadenylation and subsequent degradation of their mRNA targets[19]. 

To date, miRNAs have been assigned regulatory roles in fundamental biological processes, including differentiation, proliferation, embryonic development, and cell death [20]. Accordingly, the dysregulation of miRNA expression and function is a common observation in numerous and diverse human diseases [21]. Currently, there are over 2000 annotated mature human miRNAs, each with the capacity to regulate hundreds of target mRNAs (or approximately 30% of coding genes), establishing miRNAs as a substantial class of gene regulatory elements [22]. Importantly, miRNAs also regulate lymphocyte function and survival through both the induction and antagonism of apoptosis [23].

Previously, using both microarray and deep sequencing analysis, we described the prevalent repression of annotated miRNA expression during glucocorticoid-induced apoptosis of primary lymphocytes [1]. Further functional studies demonstrated for the first time a regulatory role for specific miRNAs and miRNA processors in the execution of glucocorticoid-induced apoptosis. Interestingly, this analysis also indicated the potential presence of numerous novel glucocorticoid-responsive miRNAs.

Here, we have developed a customized bioinformatics pipeline that facilitates the deep sequencing-mediated discovery of novel miRNAs. Using this approach, we describe the identification of hundreds of potentially novel glucocorticoid-responsive miRNAs in the transcriptome of apoptotic primary lymphocytes. Furthermore, we validated the glucocorticoid-dependent repression of two candidate novel miRNAs and Ingenuity Pathways Analysis (Ingenuity® Systems, www.ingenuity.com) predicted that these novel glucocorticoid-responsive miRNAs may contribute to glucocorticoid-induced apoptosis. In summary, these computational findings describe the discovery of novel glucocorticoid-responsive miRNAs and further suggest a potential role for both annotated and novel miRNAs in the glucocorticoid-induced apoptosis program. 

## Results

### Discovery of novel miRNAs from deep sequencing data: Generation of test and training sets

**Figure 1:**
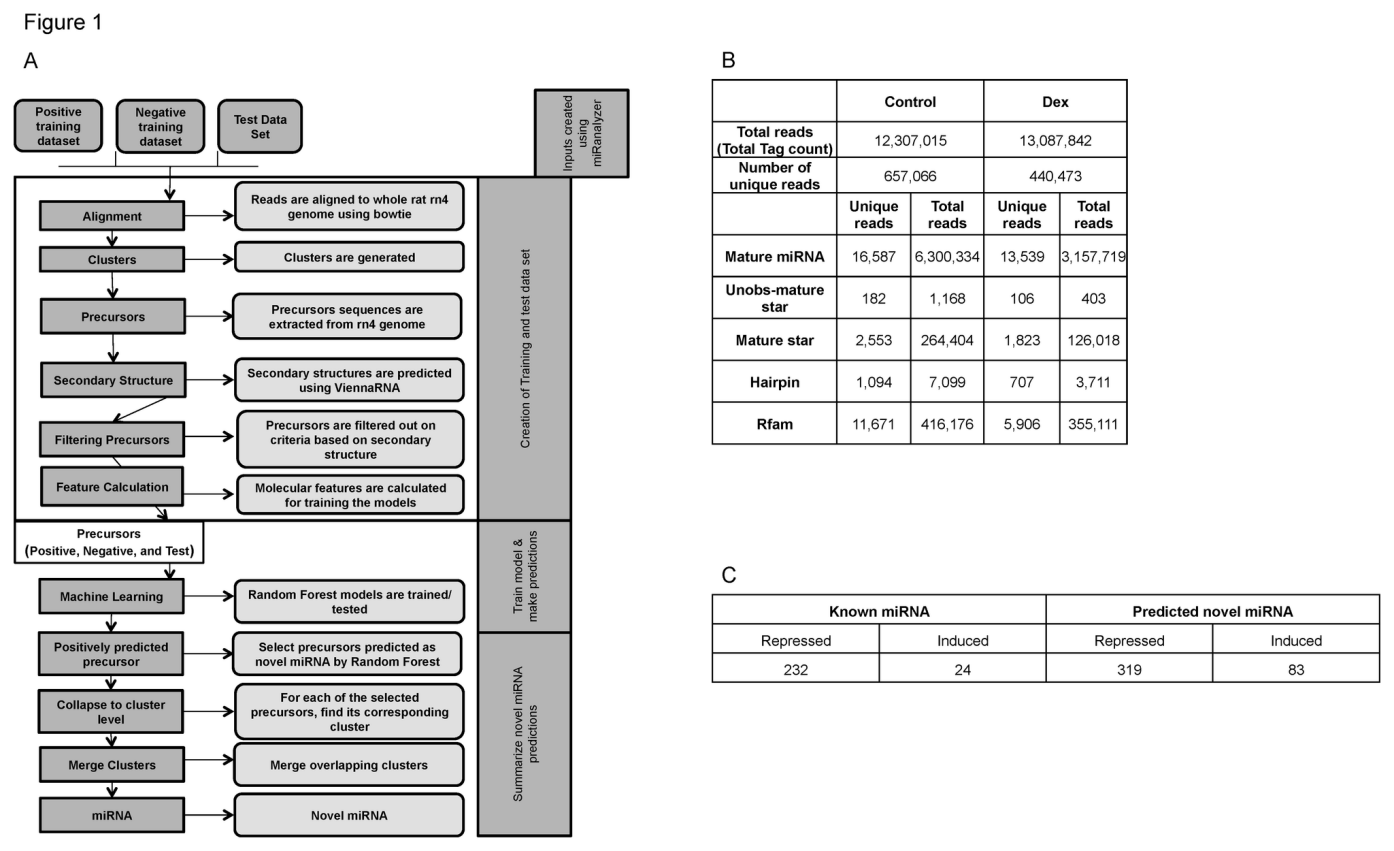
Development of a customized bioinformatics pipeline for the discovery of novel miRNAs from deep sequencing data. (A) This bioinformatics analysis workflow describes the novel miRNA discovery process adapted from miRanalyzer. The analysis pipeline uses next generation sequencing (miRNA-seq) data from untreated (control) or dexamethasone-treated rat primary thymocytes as input. This pipeline divides reads into three files: reads that align to an annotated mature miRNA (“Positive” training set), reads that align to other RNA subtypes (“Negative” training set), or reads that align at unannotated regions (“Test” set). Reads from each of these files are then aligned and alignment results are methodically processed to generate clusters, precursors and predicted secondary structures. Random forest machine learning is then employed to train the models for the prediction of novel miRNAs in the “Test” dataset. The output provides the genomic coordinates of predicted putative novel miRNAs. (B) Table describes total number of reads generated by miRNA-seq of control and dexamethasone treated primary thymocytes analyzed using the novel bioinformatics workflow described above. As expected, the majority of these reads align to known miRNAs when compared to other RNA subtypes. (C) Table summarizes the total number of known and predicted novel miRNAs identified by the bioinformatics workflow as induced or repressed in control and dexamethasone treated rat primary thymocytes. Both known and predicted novel miRNAs exhibit a trend of repressed expression during glucocorticoid-induced apoptosis.

To identify glucocorticoid-responsive novel miRNAs from deep sequencing data we employed a customized bioinformatics pipeline. This pipeline is based on miRanalyzer, a previously published methodology (also available via web-server) [24]; however, we implemented several significant modifications to the original miRanalyzer approach (see methods). The basis of this computational analysis was to first align miR-analyzer-generated reads to the genome and use ‘machine learning’ to learn from the signal profile of known miRNAs and known non-miRNAs (*training*). Once the models are trained and able to accurately classify known miRNAs from non-miRNAs, we then use the models to predict novel miRNAs from signals at unannotated regions of the genome (*testing*) (Figure 1A). 

This analysis employed reads previously generated by miRNA-seq analysis of annotated miRNAs during glucocorticoid-induced apoptosis [1]. Reads were generated by next generation sequencing on the Illumina platform using total RNA extracted from dexamethasone (Dex) treated and untreated (Control) primary thymocytes (see [1] for detailed description of apoptosis analysis). We obtained approximately 12-13 million reads for each sample and performed quality control analysis using FastQC (http://www.bioinformatics.bbsrc.ac.uk/projects/fastqc). We then trimmed all reads at the 3’ end to remove adapter sequences. Trimmed reads were subjected to a step-wise alignment protocol adopted from miRanalyzer [24] which first attempts to align reads to known miRNA sequences, and the remaining unaligned reads are then sequentially aligned to mature, mature-star*, unobserved mature-star*, hairpin, Refseq, and Rfam transcripts, sequentially (Figure S1). As a final alignment step, the remaining reads are aligned to the whole rat genome (Rn4). As expected, a large number of the total ~12-13 million reads obtained from deep sequencing of each sample aligned to known miRNAs when compared to the aforementioned RNA subtypes (Figure 1B). Reads that aligned to known miRNAs were used to generate the “Positive” training set while reads that aligned to other RNA subtypes were used to generate the “Negative” training set. Reads that did not align to any annotated RNA species but did align to the genome were used as “Test” data (Figure 1A). 

To generate sequences belonging to the “Training” and “Test” datasets, reads with overlapping genomic coordinates were grouped together to form ‘clusters’ (totaling a length of 20-27 nucleotides) and several ‘precursor’ sequences were generated from each cluster. Precursors encompassed a genomic window centered at the cluster and extending on both the 5’ and 3’ ends of the cluster. We obtained 284 Control and 236 Dex clusters in the true “Positive” and 5,499 Control and 3,179 Dex clusters in the true “Negative” training data (Table S1A). Generated precursor sequences were then subjected to secondary structure selection criteria (Figure 1A).

 The secondary structure of each precursor sequence was generated using Vienna RNA [25] and the precursor sequence was discarded from further consideration if the secondary structure did not meet stringent criteria. Pre-miRNAs are characterized by a canonical stem loop structure, hence the selection criteria was designed to discard all precursors whose secondary structure did not exhibit the desired number of base pairing and a stable hairpin structure. The filtered precursor sequences that met these criteria were used to generate molecular features that describe the unique sequence and/or secondary structure attributes of the precursor candidate in question. We chose a set of ten molecular features that best characterize attributes distinguishing a miRNA from other RNA subtypes (Figure 1A). These include features that characterize the degree of conservation of the miRNA sequence, the signal intensity at each putative miRNA location, and characteristics of the predicted secondary structure including the minimum free energy (Figure S2). 

### Training of random forest models

We generated molecular features for all filtered precursor sequences within the “Positive” and “Negative” training sets and constructed random forest models for both the Control and Dex datasets (Figure 1A). While our modeling technique used information from all molecular features for classification purposes, our analysis indicated that certain molecular features had more influence on the classification. The sequence conservation score was the most informative feature whereas the number of bases in the overhang of the secondary structure was among the least informative (Figure S2). The training classification error for random forest models ranged from 93.3% to 99.8% denoting a high degree of accuracy (Table S2A and S2B). 

### Prediction of novel miRNAs using trained models

The “Test” dataset was processed in a manner identical to the “Training” dataset in terms of preparation of clusters and precursor sequences; however, we eliminated clusters from further analysis if miRNA expression signal was below 11 raw read count to focus on only those miRNAs displaying moderate to high expression levels. We obtained 15,332 Control and 9,876 Dex clusters resulting in 52,354 Control and 33,646 Dex precursors in the “Test” dataset (Table S1B). These precursors were subjected to classification using our modeling technique. 

The precursor sequences that were predicted as novel miRNAs were further filtered based on two criteria: (i) the minimum free energy of their predicted secondary structures, and (ii) signal intensity. This yielded 515 and 346 novel miRNAs predicted for Control and Dex samples, respectively, with 220 common between the two sample types. Previously, we reported that the majority of known miRNAs are repressed during glucocorticoid-induced apoptosis of lymphocytes [1]. Interestingly, this trend extends to our analysis of miRNA-seq-derived novel miRNAs. Here, approximately 80% of predicted novel miRNAs were repressed in response to dexamethasone treatment (Figure 1C).

### Validation of novel glucocorticoid-responsive miRNAs

**Figure 2:**
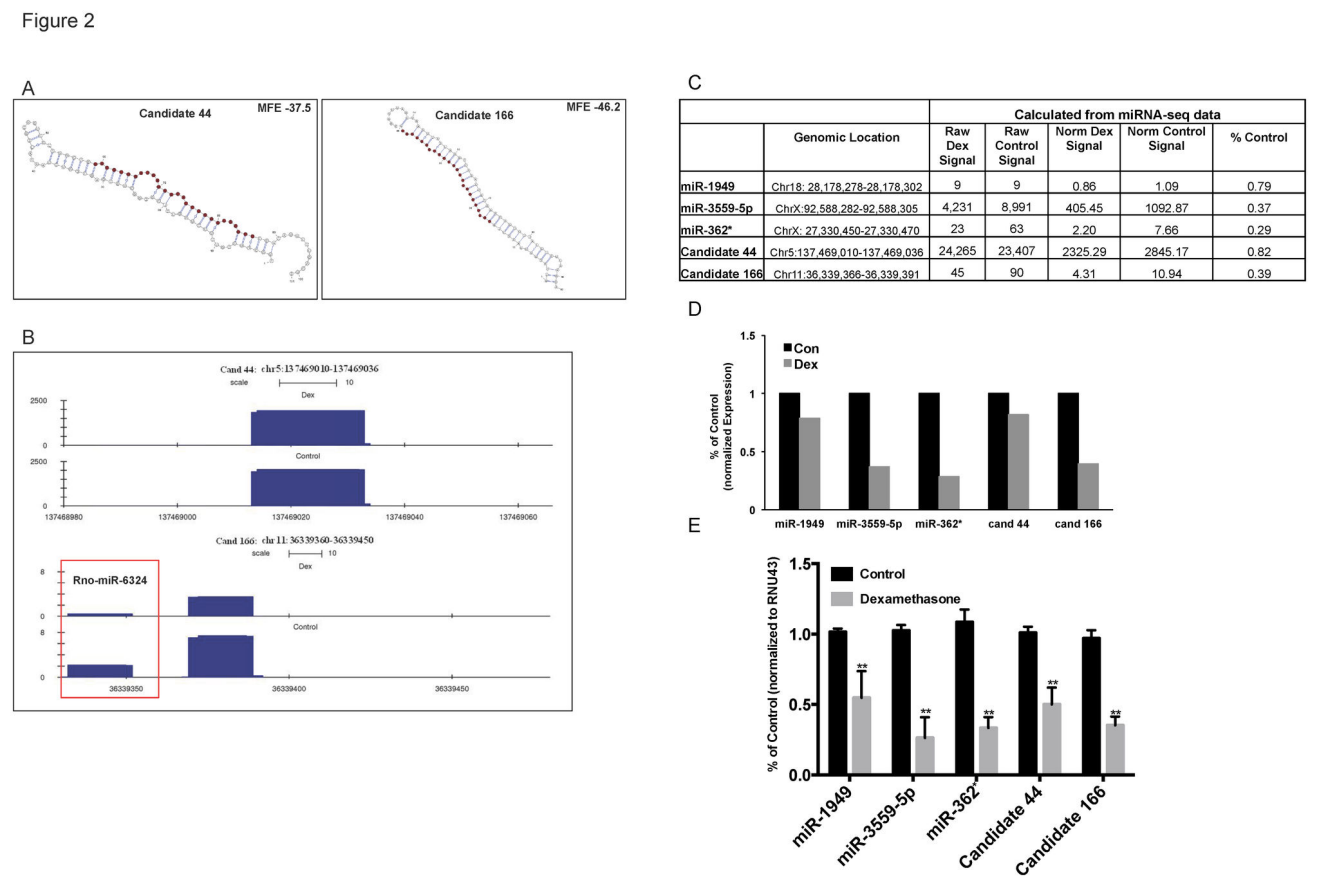
Validation of novel glucocorticoid responsive miRNAs. (A) Secondary structure of two novel miRNA generated by ViennaRNA. The predicted ‘mature’ sequence is highlighted in red; the remaining hairpin contains the putative stem loop and mature-star* sequence, the minimum free energy (MFE) of each structure is indicated. The VARNA visualization applet was used to draw the RNA secondary structure [66]. (B) The expression of the candidate novel miRNAs, candidates 44 and 166 visualized in UCSC genome browser (Dex treated is top bar, Control is bottom bar). Both of the predicted novel miRNAs are repressed during glucocorticoid-induced apoptosis of primary lymphocytes. Visualization of novel miRNA candidate 166 also detects a glucocorticoid-responsive signal at the proximal newly annotated mature miRNA rno-miR-6324, which is antisense to candidate 166 (indicated in the red box). (C) Percent control values of the five miRNAs (3 known and 2 predicted novel candidates) selected for qPCR validation. Percent control was calculated as (Dex/Control) using computationally derived signal values for control and dexamethasone-treated rat primary thymocytes. Signal values were generated using stringent sequence alignment criteria of miRNA-seq data. (D) Graphic representation of percent control values for control and dexamethasone-treated samples generated using computationally derived expression signals from the miRNA-seq data. Raw read counts at each miRNA were normalized to the total number of aligned reads in the respective sample to generate normalized signal. (E) Rat primary thymocytes were untreated (control) or treated with 100nM dexamethasone for 6 hours (apoptosis was monitored as previously described [1]). The expression of annotated positive controls and individual mature candidates was evaluated via quantitative PCR using custom TaqMan Small RNA Assays. The expression of RNU43 small nuclear RNA served as an endogenous control. Results are reported as mean percent control values +/- SEM values for 3 biological replicates (**p<.01).

To verify the glucocorticoid-induced repression of miRNAs, a combination of both annotated and novel miRNA candidates were selected for qPCR validation. Two novel miRNA candidates, candidate 44 and candidate 166, were chosen for validation on the basis of their predicted secondary structure. Both candidates demonstrate a canonical stem-loop structure and a putative mature miRNA sequence (Figure 2A). Furthermore, the expression of each novel miRNA candidate (as visualized in the UCSC Genome Browser [26]) is repressed in response to dexamethasone treatment (Figure 2B). This observation parallels the trend of prevalent repression of annotated miRNAs during glucocorticoid-induced apoptosis of lymphocytes, suggesting that these novel candidates are biologically similar to annotated miRNAs. Interestingly, candidate 166 also exhibits detectable signal at the proximal mature miRNA rno-miR-6324, a recently annotated mature miRNA arising from the same precursor as candidate 166 [27]. While the basal expression of rno-miR-3624 is lower than candidate 166, it is also repressed in response to dexamethasone treatment (Figure 2B). 

A total of five candidates, three annotated miRNAs (miR-1949, miR-3559-5p, and miR-362*) and the two predicted novel miRNAs (candidates 44 and 166) were subjected to small-RNA qPCR analysis. Each validation candidate exhibited sufficient basal signal for qPCR analysis and a degree of glucocorticoid-responsiveness as determined by the percent control value generated from computationally derived expression signals (Figure 2C and 2D). Custom Taqman Small RNA assays were designed to the mature 5’-3’ sequence of each candidate miRNA and used for the targeted quantitation of novel glucocorticoid-responsive miRNAs. These assays employ a sequence-specific stem-loop 3’ reverse transcription primer, thereby assuring the definitive analysis of small RNAs [28]. This analysis confirmed the significant repression of both the annotated positive controls as well as the novel candidate miRNAs during glucocorticoid-induced apoptosis of primary lymphocytes (Figure 2E). Interestingly, the percent of control values generated by qPCR analysis closely mirror those derived from the miRNA-seq data (Figure 2D). These findings confirm the presence of two and predict the existence of numerous additional novel glucocorticoid-responsive miRNAs in the rat transcriptome (Figure 1C). To explore the potential functional roles of these novel glucocorticoid-responsive novel miRNAs, we performed further computational analysis to identify the predicted gene targets for each of the two qPCR-validated novel miRNAs. 

### Pathways analysis predicts novel miRNA targets may contribute to glucocorticoid-induced apoptosis

**Figure 3:**
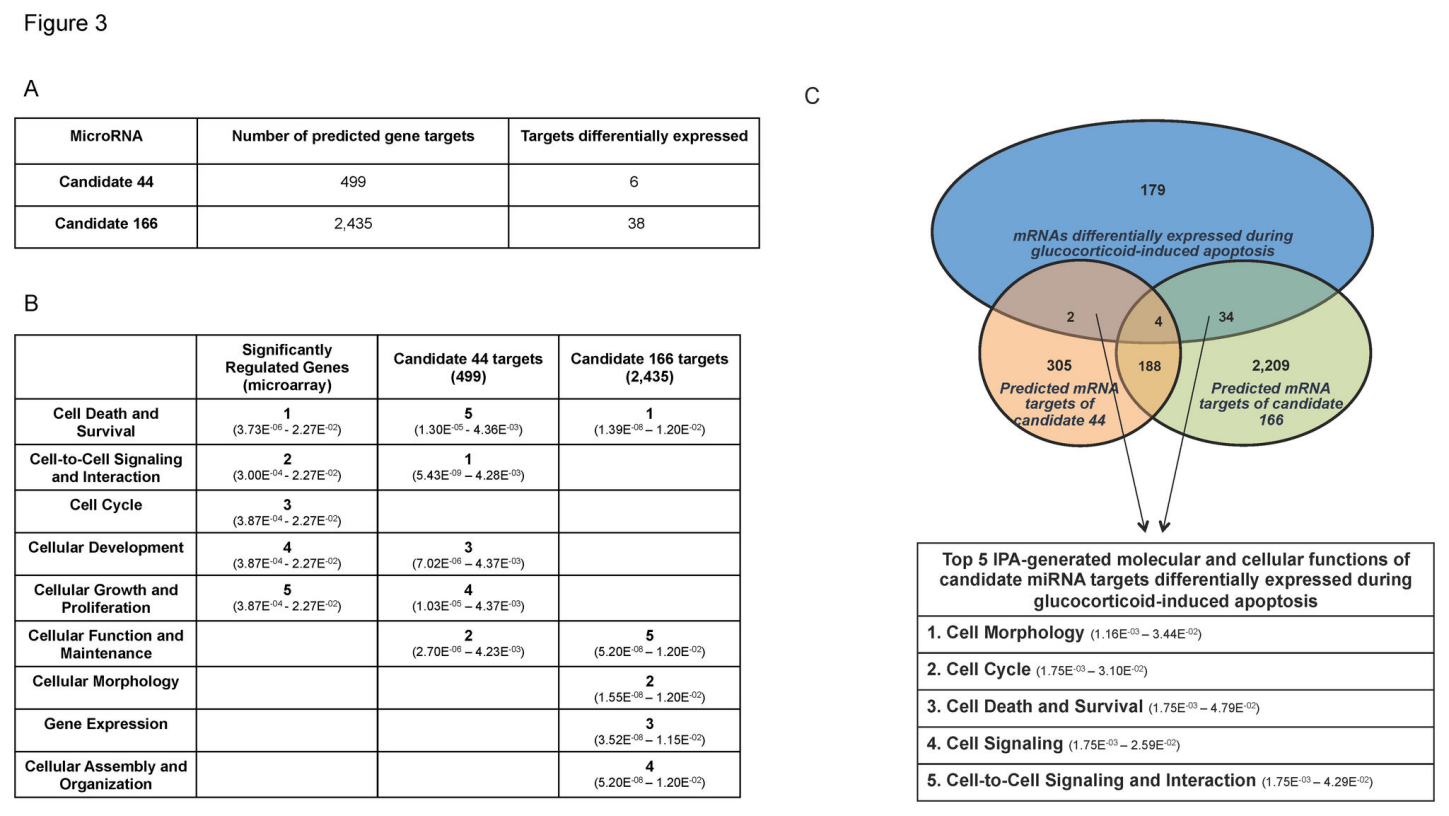
Pathways analysis predicts novel miRNA targets may contribute to glucocorticoid-induced apoptosis. (A) miRNA target predictions for novel miRNA candidates 44 and 166 were performed using the miRanda miRNA target prediction algorithm. The number of target mRNAs differentially expressed during glucocorticoid-induced apoptosis (p < 0.01; fold change > 1.2) is indicated for each candidate. (B) IPA-generated ranking of the top five molecular and cellular functions of genes differentially expressed during glucocorticoid-induced apoptosis (p < 0.01; fold change > 1.2), as well as the predicted targets of both candidates 44 and 166 (p-values for top functions are indicated beneath each ranking). Genes differentially expressed during glucocorticoid-induced apoptosis were identified by whole genome microarray analysis of untreated and 100nM dexamethasone-treated thymocytes (6 hours, 3 biological replicates). (C) Venn diagram analysis identified specific novel candidate predicted targets differentially expressed during glucocorticoid-induced apoptosis (p<.01) and the application IPA to this combined gene list (40 genes) generated a top 5 ranking of molecular and cellular functions regulated by these predicted targets (p-values for top functions are indicated beneath each ranking).

Using the mature sequence of novel miRNA candidates 44 and 166, gene target predictions were made against the 3’ untranslated regions of RefSeq transcripts via the miRanda miRNA target prediction algorithm [29]. Numerous gene targets were predicted for both candidate novel miRNAs (Figure 3A). To assess the potential role of these predicted targets in the glucocorticoid-induced apoptosis program, whole genome gene expression microarray was performed on both untreated and dexamethasone treated primary thymocytes (3 biological replicates each). Ingenuity Pathways Analysis (IPA) of genes deemed differentially expressed (p-value < 0.01 and absolute fold change > 1.2) suggests that they govern molecular and cellular functions involving cell proliferation, cell division, and cell death (Figure 3B). Interestingly, IPA of the predicted novel miRNA targets suggests that these miRNAs may contribute to many of the same molecular and cellular functions identified by the whole genome microarray analysis. Specifically, cell death and cell survival is a top IPA-generated molecular and cellular function for the miRanda predicted targets of both candidates 44 and 166 (Figure 3B). 

Further Venn diagram analysis identified specific mRNA targets of candidates 44 and 166 differentially expressed during glucocorticoid-induced apoptosis (Figure 3A). IPA of this combined gene list identified cell death and survival as a top predicted molecular and cellular function of these differentially expressed potential targets, as well as other functions critical to the induction and execution of glucocorticoid-induced apoptosis, including changes in cell morphology, cell cycle and cell signaling (Figure 3C). These computational findings suggest that these novel glucocorticoid-responsive miRNAs may contribute to glucocorticoid-induced apoptosis.

## Discussion

Previously, using both microarray and deep sequencing analysis, we described the prevalent repression of annotated miRNAs during glucocorticoid-induced apoptosis of primary rat thymocytes [1]. Additional studies have demonstrated the glucocorticoid-mediated regulation of specific miRNAs in lymphoid cells, and further delineated a functional role for these miRNAs in the execution of glucocorticoid-induced apoptosis [30-33]. For example, studies by both Harada et al. and Molitoris et al. report the glucocorticoid-mediated repression of the miR-17 family, resulting in increased Bim expression, and, consequently, increased sensitivity to glucocorticoid-induced apoptosis [31,32]. Alternatively, several studies have reported that specific miRNAs regulate glucocorticoid sensitivity and contribute to glucocorticoid-resistance in lymphoid malignancies [34-37]. 

In our present study, we propose the existence of novel, unannotated, glucocorticoid-responsive miRNAs with expression profiles similar to those we previously described for annotated miRNAs (dexamethasone-induced repression). Given that deep sequencing technology provides a powerful, unbiased platform to measure the expression of miRNAs we sought to further explore and catalogue the presence of novel glucocorticoid-responsive miRNAs in the rat transcriptome. To this end, we developed a bioinformatics pipeline combining elements of miRanalyzer [24], a peer-reviewed publically available miRNA discovery approach, and a customized machine learning technique to facilitate the identification of novel miRNAs from deep sequencing data. 

 The discovery of novel miRNAs from deep sequencing data is a rapidly expanding area of bioinformatics research. To date, numerous studies have reported the deep sequencing-mediated discovery of novel miRNAs in diverse systems including viruses [38,39], plants [40-43], insects [44], lower vertebrates [45,46], mammals [47,48], cell culture [49,50], and human patient samples [51-55]. Interestingly, several of these studies report the altered expression profile of these newly identified miRNAs during pathophysiological conditions including aging, Sjogren’s Syndrome, psoriasis, b-cell malignancy, and lung cancer [48,51-54]. Our study extends these findings to non-transformed, mammalian primary lymphocytes and, to our knowledge, is the first to report the hormonal-regulation of novel miRNA expression. Importantly, the recent, independent discovery of rno-miR-6324 (a mature miRNA in the anti-sense orientation to candidate 166) strengthens the evidence that candidate 166 is a novel, glucocorticoid-responsive miRNA and that our approach to the identification of novel miRNAs from deep-sequencing data is both accurate and reproducible [27].

 We next employed IPA to characterize the potential cellular and molecular functions of the newly validated glucocorticoid-responsive miRNAs. This analysis indicated that the putative targets of these novel miRNAs are predicted to influence cell death. Pathways analysis of specific novel miRNA candidate targets differentially regulated during glucocorticoid-induced apoptosis identified cell death and survival as a top-regulated predicted cellular and molecular function as well as other cellular processes essential for the glucocorticoid-induced cell death program, including changes in cellular morphology, cell cycle, and cellular signaling [56]. Presently, further functional analyses of these novel miRNAs in this model system are not possible, since rat primary thymocytes are not amenable to genetic manipulation in-vitro. However, these preliminary IPA-derived functional predictions provide a promising basis for the future validation and functional analysis of both novel miRNAs in an alternative, adaptable model system.

 In summary, these studies employ a customized bioinformatic pipeline that enables the discovery of novel miRNAs from deep sequencing data and further describes the repression of two novel miRNAs (candidates 44 and 166) during glucocorticoid-induced apoptosis of primary thymocytes. Computational analysis predicts that miRNA candidates 44 and 166 may contribute to the glucocorticoid-induced apoptosis program through the regulation of target mRNAs involved in cell death and survival functions. These findings are the first to identify the presence of novel, glucocorticoid-responsive miRNAs in the rat transcriptome.

## Materials and Methods

### Ethics Statement

All animal experiments were approved by the National Institute of Environmental Health Sciences Institutional Animal Care and Use Committee and complied with USDA Column C classification (minimal, transient, or no pain or distress). Experimental animals were routinely monitored by NIEHS veterinary staff and investigators for pain or distress.

### Rat primary thymocyte isolation

Rat primary thymoyctes were isolated from adrenalectomized (60-75g) male Sprague-Dawley rats (Charles River Laboratories, Wilmington, MA) approximately 1-2 weeks after surgery. Following decapitation, the thymi of three animals were removed and pooled in RPMI 1640 medium containing 10% heat-inactivated fetal bovine serum, 4 mM glutamine, 75 units/ml streptomycin, and 100 units/ml penicillin. Thymi were gently sheared with surgical scissors at room temperature. Sheared cells were filtered through 200-micron nylon mesh twice and centrifuged at 3K for 5 minutes at room temperature. The cell pellet was then resuspended in fresh media and filtered into a sterile conical tube. Cells were cultured at a final concentration of 2x10^6^ cells/mL and incubated at 37°C, 5% CO_2_ atmosphere. 

### miRNA deep sequencing

Rat primary thymocytes were isolated and cultured in the presence or absence of 100nM dexamethasone for 6 hours. Following treatment, total RNA was isolated using the Ambion mirVana miRNA isolation kit (Austin, TX) from untreated control and dexamethasone-treated samples and subjected to miRNA Deep Sequencing. Small RNA cDNA libraries were prepared according to manufacturer’s protocol (Small RNA Sample Prep Kit Oligo Only, protocol 71003, Illumina, Inc., San Diego, CA). Small RNA cDNA libraries were then sequenced according to manufacturer’s instructions on the Illumina Genome Analyzer II (Illumina, Inc., San Diego, CA). The data discussed in this publication have been deposited in NCBI's Sequence Read Archive [57] and are accessible through SRA accession number SRP019941.

### Bioinformatic analysis of miRNA deep sequencing data

Deep sequencing data for one lane each of Dex and Control samples were received in the fasta format. Read lengths of Dex samples was 35 nucleotides whereas for control it was 25 nucleotides. However approximate length of a mature miRNA is around 18-22 nucleotides therefore it is likely that the 3’ end of the read sequence may contain adapter sequences. To remove possible adapter sequences we trimmed the reads at 3’ end such that resulting reads were 20 nucleotides in length. Next, the sequence reads were collapsed into a fasta formatted file where only unique sequences remain and duplicated sequences were counted and recorded in the header information for each sequence. Out of 13,087,842 and 12,307,015 reads in Dex and Control respectively, the data was compressed to 440,473 and 657,066 unique reads respectively. The resulting files were used for further analysis, which included discovery of novel miRNAs and calculation of differential expression in Dex vs. Control for novel and existing miRNAs. 

### Computational prediction of novel miRNAs

To discover novel miRNAs from deep sequencing data, we designed a bioinformatics pipeline based on the miRanalyzer methodology. miRanalzyer is a web server that uses input short sequence reads of lengths up to 25nts and outputs predicted novel miRNAs [24]. It is also available in a stand alone version [58]. Moreover, our experimentation with the software determined that the implementation of the random forest prediction approach within miRanalyzer was not robust enough to yield reproducible results. To overcome these limitations we implemented a number of new ideas within the novel miRNA discovery paradigm. To this end, we designed a data analysis workflow that uses the general framework and certain components from miRanalyzer and combines it with our novel machine learning approach. 

First, we implemented a sequence alignment strategy as described in miRanalyzer. The fasta files from Dex and Control samples were used as input. Alignments were performed in a sequential manner. First, reads were aligned to known miRNAs, followed by alignment to mature-star*, mature-star* unobserved and hairpin precursors. Next, the remaining reads were aligned to known mRNAs and RNA families as defined by RFAM. The sequences that map to any of the above RNA subtypes are then removed. Remaining sequences are aligned to the Rn4 genome (Figure S1). 

Reads aligning to mature miRNAs were used to build the true “Positive” training dataset and reads aligned to other RNA types such as RFAM was used to build “Negative” training dataset. Reads that did not map to any known RNAs but map to unannotated locations in the genome were used to build the “Test” dataset. The alignments for training/test dataset were generated using bowtie (0.12.7) [59] with –best and –strata options. We allowed up to 2 mismatches in the seed length of 17 and up to 6 alignments were allowed per read. Only the longest alignments that maintained the number of observed mismatches within the seed were kept for further analysis.

Following the miRanalyzer approach, all overlapping aligned reads were grouped together and ‘clusters’ were formed. ‘Precursor’ sequences were then generated from each cluster [58]. We predicted the secondary structure of each precursor sequence using the ViennaRNA (version 2.0.6) [60] tool and removed precursors if any of the following were true:

1. It doesn’t have single stem hairpin structure
2. If it has less than 19 bindings to the candidate precursor sequence
3. If it has less than 11 bindings to the region occupied by the read cluster
4. If candidate precursor genomic location doesn’t overlap with a known miRNA (only in case of true positive data set)

For the remaining precursor sequences, we calculated the molecular attributes that best describe the sequence and secondary structure characteristics of the precursor sequences. These characteristics are then used as input to the machine learning methods to train the models. The molecular features used include:

1. Total number of bindings within the read cluster
2. Total number of bindings in whole candidate precursor secondary structure
3. The length of the read cluster
4. The expression of mature-star* sequence
5. Total tag counts in the read cluster
6. The minimum free energy (MFE)
7. Normalized Energy (MFE/candidate precursor length)
8. The difference in the number of nucleotides that don’t bind between the arms
9. The expression of overlapping conserved region
10. The number of unbinding nucleotides in overhang region

Using these features calculated for each precursor sequence in the positive and negative training dataset, we built two random forest models, one each for the Control and Dex data using “randomForest” R-Package [61]. The random forest model consisted of 1000 binary decision trees, each constructed from 66% of randomly selected training precursors and 3 randomly selected training features. For each training sample, aggregated classification votes were computed from all the trees in which the sample under consideration was excluded. Next, the out of bag training error/accuracy rates were computed, using above classification vote counts. The importance of each of the training feature is assessed using change in out of bag training accuracy, after permuting the values of feature of interest (Figure S2 shows the ranking of features). Ranking is calculated by mean decrease in accuracy associated with each feature.

Our training models displayed significantly high classification accuracy (i.e. low class error) as described by the confusion matrix (Table S2). We employed 1000 trees for modeling, a significantly large number compared to the miRanalyzer, to ensure that training and testing results are consistent and reproducible. 

We used reads from the “Test” dataset and generated clusters and precursor sequences as described earlier. Here, we discarded clusters with raw read counts (expression value) lower than 11 prior to precursor generation step to avoid regions with low expression. The resulting precursor sequences from the test data were used for feature generation and as input for classification using the two random forest models trained from the Control and Dex data as described earlier. 

Precursor sequences that were predicted to be novel miRNAs were identified, and the parent ‘cluster’ sequence for each of those precursors was used as the novel ‘mature’ miRNA sequence. These novel miRNAs were further discarded if they met either of the following criteria:

1. If the predicted novel miRNA localizes to chrUn or any of the ‘random’ chromosomes.
2. If the MFE of predicted novel miRNA is greater than -25.

In cases where the chromosomal coordinates of the novel miRNAs overlapped each other, they were merged to form one novel miRNA. 

### miRNA signal and differential expression calculation

To determine computationally derived expression values at each annotated and predicted novel miRNA, we counted the total number of reads aligned at a genomic locus normalized by the total aligned reads for a given sample (Reads Per Million). For this calculation, we only included those reads that met very stringent sequence alignment criteria (reads may have a maximum of 3 alignments and only one mismatched position within the 17nt seed length). To determine whether a given miRNA is induced or repressed in response to dexamethasone treatment, we calculated the ratio of signal in Dex divided by Control. If the ratio is above 1, we consider the miRNA induced by Dex, if the ratio is below 1, we consider the miRNA repressed by Dex. 

### Novel miRNA qPCR

Total RNAs were isolated from control and dexamethasone-treated (100nM, 6 hours) thymocytes using the Ambion mirVana miRNA isolation kit (Austin, TX). For annotated and novel miRNA validations, total RNAs were reverse transcribed using the Taqman miRNA Reverse Transcription kit (Applied Biosystems, CA, USA) and analyzed using custom-designed Taqman Small RNA Assays (Applied Biosystems, CA, USA) per manufacturer instructions. Single-tube primer/probes for each candidate were designed using the Custom TaqMan Small RNA Assay Design Tool using the predicted (or annotated) mature miRNA sequence as the design template. Prior to submission, template sequences were evaluated for specificity via the Basic Local Alignment Search Tool (BLAST) [62]. Primer template sequences for each candidate novel miRNA were: 

Candidate 44: CGCGGATGATGACACCTGGGTAT

Candidate 166: GCTCTGCTGACTGCCTATGGGCT

Each customized small RNA assay was evaluated for signal in both reverse transcriptase minus and the cDNA minus non-template controls, indicating the detection of small-RNA-specific signal. Each primer/probe was normalized to the expression of the small-nucleolar RNA RNU43. 

### Whole genome microarray

Rat primary thymocytes were isolated and cultured in the presence or absence of 100nM dexamethasone for 6 hours. Following treatment, total RNA was isolated from three biological replicates using the Ambion mirVana miRNA isolation kit (Austin, TX) and subjected to whole genome microarray analysis. Gene expression analysis was conducted using Agilent Whole Rat Genome 4x44 multiplex format oligo arrays (014879) (Agilent Technologies) following the Agilent 1-color microarray-based gene expression analysis protocol. Starting with 500ng of total RNA, Cy3 labeled cRNA was produced according to manufacturer’s protocol. For each sample, 1.65ug of Cy3 labeled cRNAs were fragmented and hybridized for 17 hours in a rotating hybridization oven. Slides were washed and then scanned with an Agilent Scanner. Data was obtained using the Agilent Feature Extraction software (v9.5), using the 1-color defaults for all parameters. The Agilent Feature Extraction Software performed error modeling, adjusting for additive and multiplicative noise. The resulting data were processed using the Rosetta Resolver® system (version 7.2) (Rosetta Biosoftware, Kirkland, WA). The data discussed in this publication have been deposited in NCBI's Gene Expression Omnibus [63] and are accessible through GEO Series accession number GSE45560.

### Analysis of whole genome microarray data

The feature extractor processed raw signal was log_2_-transformed, quantile normalized and summarized for each probe using median polish algorithm. Next, we identified differentially expressed genes in Dex treated compared to Control samples using signal to noise statistic defined as the ratio of average signal difference and sum of between replicate standard deviations. The adjusted and unadjusted p-values for this signal to noise statistic was computed using left/right tail of empirical distribution generated by 10,000 sample/probe permutations (similar to [64]. We used a nominal p-value threshold of 0.01 (nominal p-value =< 0.01) and absolute fold change threshold of 1.2 (absolute fold >= 1.2) to identify differentially expressed probes. We used available probe annotation to map probe IDs to corresponding RefSeq genes. We identified 219 genes with statistically significant differential expression.

### Prediction novel miRNA targets

Prediction of gene targets for a given miRNA was conducted using miRanda software [65]. We used the mature miRNA sequence as an input to the program and the software generated predicted gene targets by comparing complementarity in the seed region of the miRNA sequence to the 3’ UTR sequence of all known mRNAs in the genome. The list of gene targets for each of the two candidate novel miRNA was further analyzed for enrichment of biological pathways using IPA.

### Pathway analysis using IPA

We employed Ingenuity Pathway Analysis software to identify enriched biological pathways and molecular functions within a given gene list. We performed IPA on a list of gene targets for each of the two candidate novel miRNAs (candidate 44 and 166), and also performed IPA on the list of differentially expressed genes identified by the microarray analysis. IPA was also performed on the subset of gene targets, as identified by the Venn Diagram analysis, to be differentially expressed in the microarray analysis (Figure 3). 

## Supporting Information

Figure S1 **Alignment workflow based on original miRanalyzer.** Work-flow diagram of sequence alignment as implemented in miRanalyzer. The figure was adapted from the miRanalyzer manuscript [58].
(TIFF)

Figure S2 **Accuracy of molecular features used in computational prediction of miRNAs.** Figure displays ranking of molecular features used in computational prediction of miRNAs. The x-axis reports the mean decrease in accuracy of the model for each of the molecular features in question. Conservation is the most informative feature in this analysis.
(TIFF)

Table S1 **Summary of training and test data sets.** (A) Table describes number of unique clusters and resulting precursor sequences for Dex and Control samples in positive and negative training sets.
(B) Table describes number of unique clusters and resulting precursor sequences for Dex and Control samples in test set.
(TIFF)

Table S2 **Confusion matrix for training data for control and dexamethasone-treated thymocytes.** (A and B) Confusion matrix displaying predicted and actual number of microRNAs and non-microRNAs as identified by our computational analysis. The classification accuracies of predicting microRNAs are listed for both control and dexamethasone-treated samples.
(TIFF)
