# Supplementary figures and images for: Deep Sequencing Identification of Novel Glucocorticoid-Responsive miRNAs in Apoptotic Primary Lymphocytes
Source: PLoS One. 2013 Oct 24;8(10):e78316. doi: 10.1371/journal.pone.0078316 (PMC3824063; doi:10.1371/journal.pone.0078316)

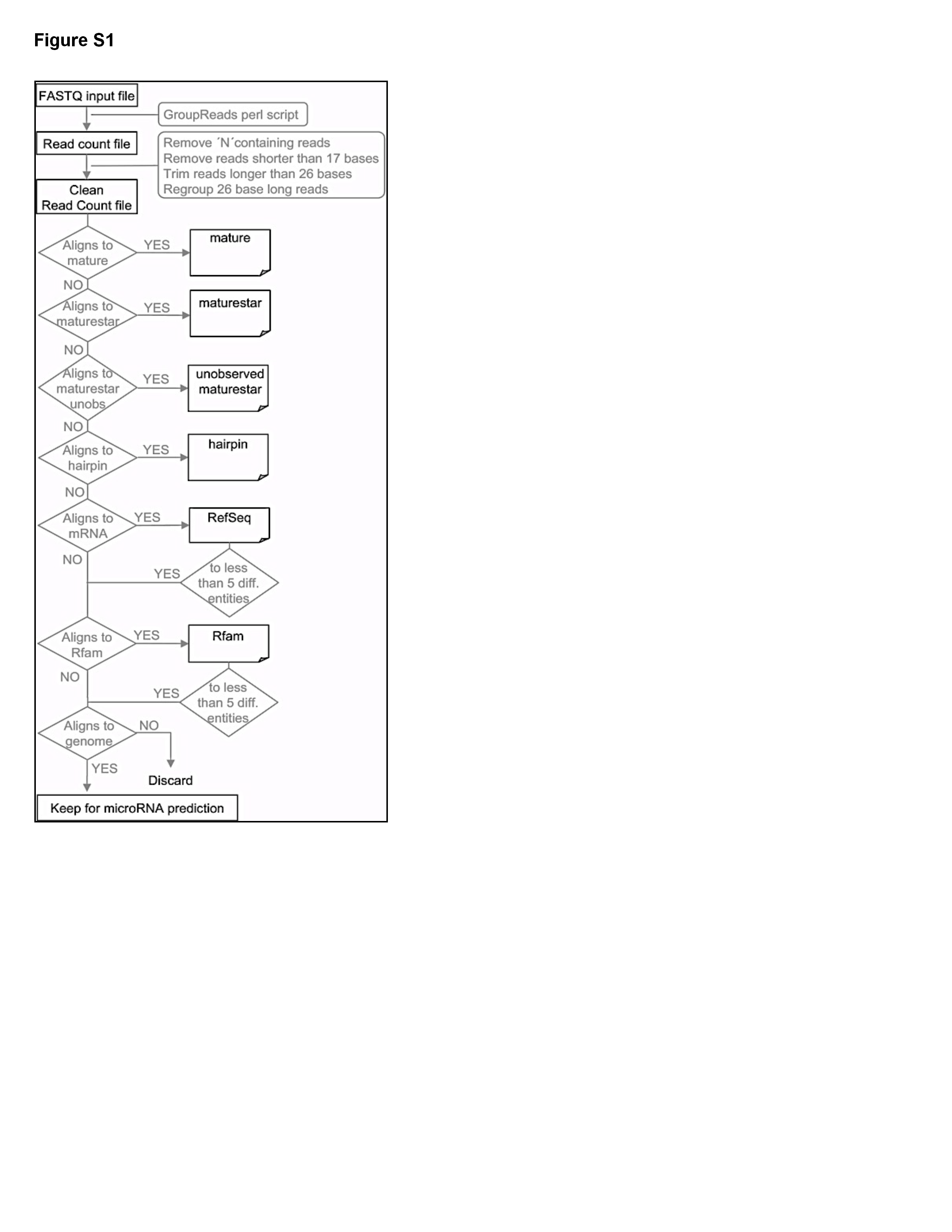

Supplement: Figure S1 — Alignment workflow based on original miRanalyzer. Work-flow diagram of sequence alignment as implemented in miRanalyzer. The figure was adapted from the miRanalyzer manuscript [58]. (TIFF) [file pone.0078316.s001.tiff]

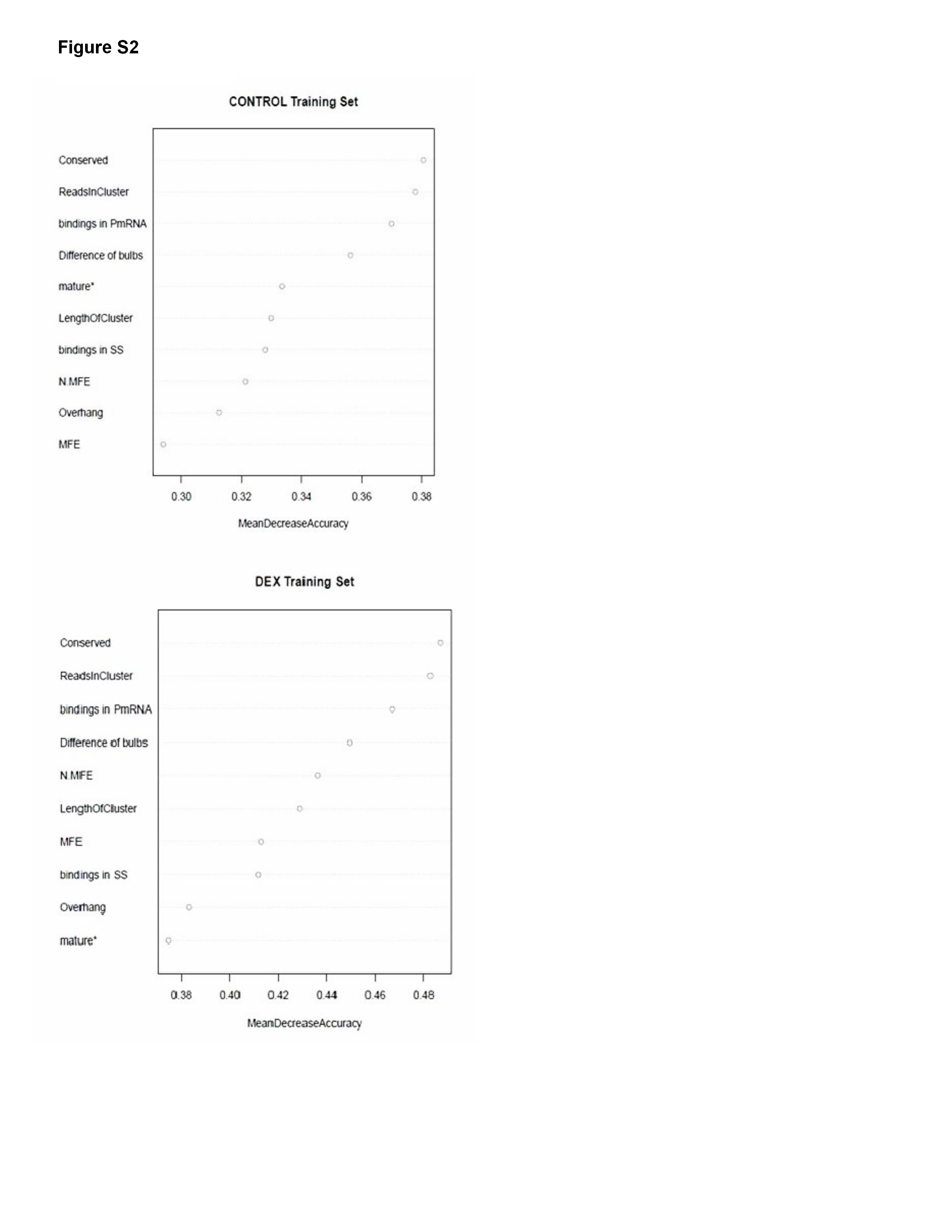

Supplement: Figure S2 — Accuracy of molecular features used in computational prediction of miRNAs. Figure displays ranking of molecular features used in computational prediction of miRNAs. The x-axis reports the mean decrease in accuracy of the model for each of the molecular features in question. Conservation is the most informative feature in this analysis. (TIFF) [file pone.0078316.s002.tiff]

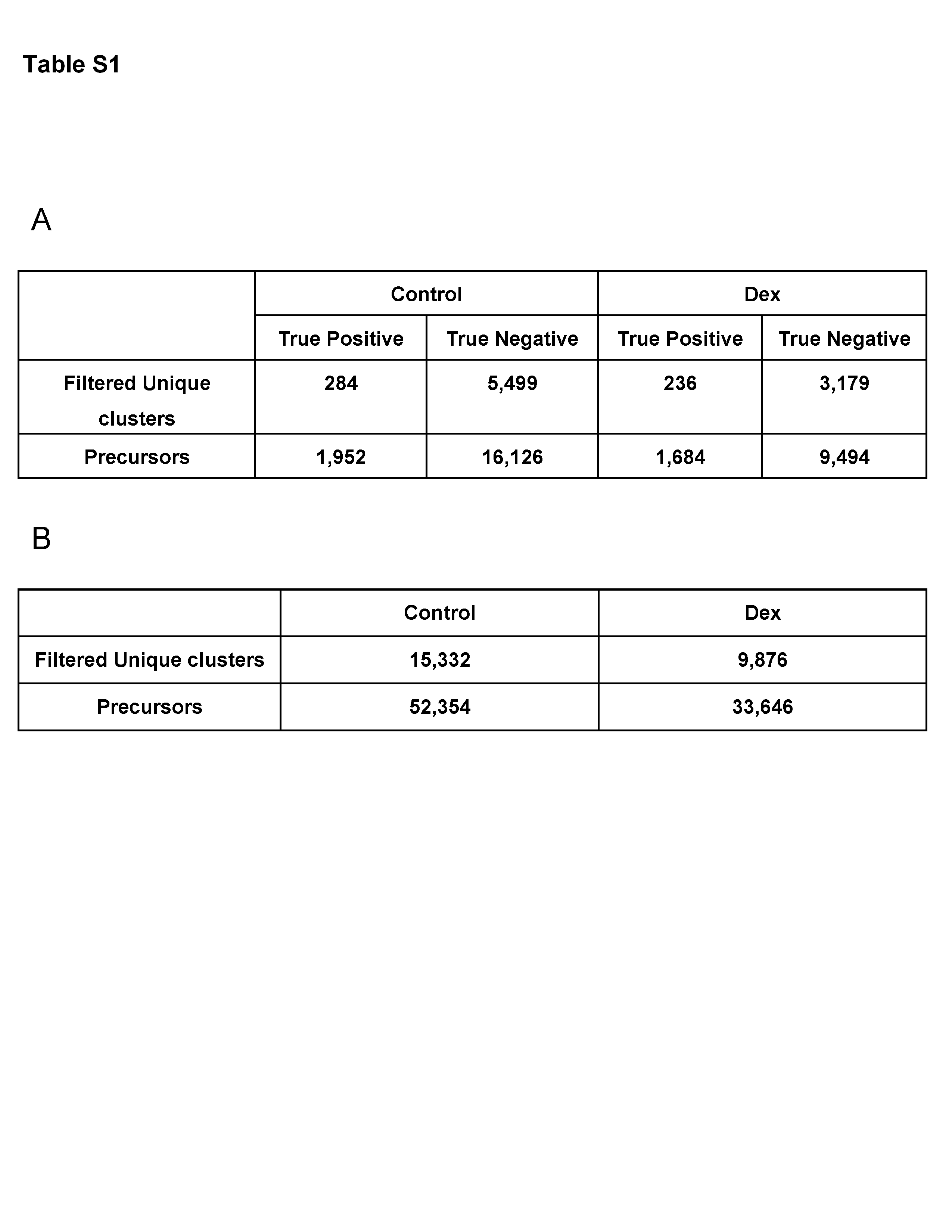

Supplement: Table S1 — Summary of training and test data sets. (A) Table describes number of unique clusters and resulting precursor sequences for Dex and Control samples in positive and negative training sets. (B) Table describes number of unique clusters and resulting precursor sequences for Dex and Control samples in test set. (TIFF) [file pone.0078316.s003.tiff]

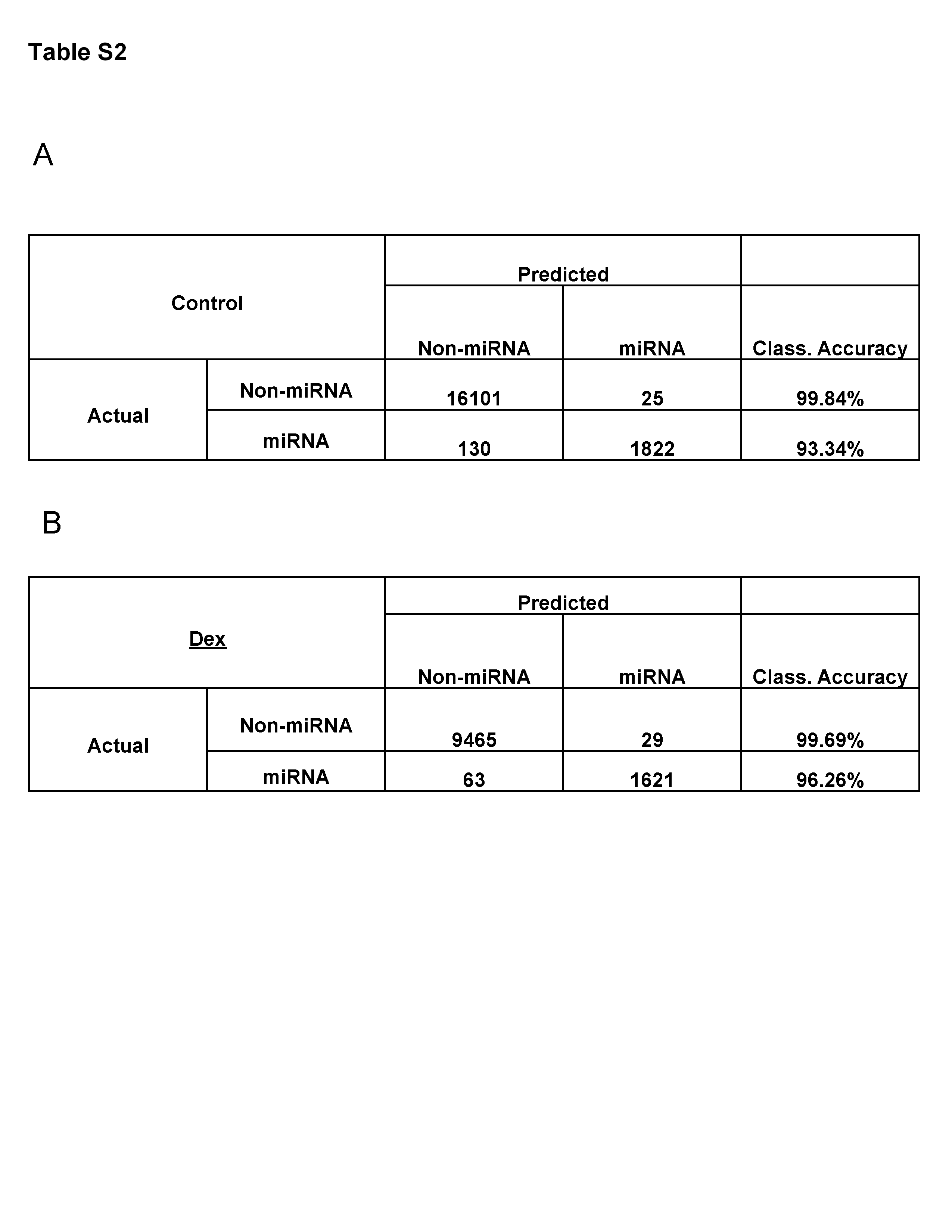

Supplement: Table S2 — Confusion matrix for training data for control and dexamethasone-treated thymocytes. (A and B) Confusion matrix displaying predicted and actual number of microRNAs and non-microRNAs as identified by our computational analysis. The classification accuracies of predicting microRNAs are listed for both control and dexamethasone-treated samples. (TIFF) [file pone.0078316.s004.tiff]
